# Supplementary material for: Molecular characterization of carbapenem-resistant Klebsiella pneumoniae isolates with focus on antimicrobial resistance
Source: BMC Genomics. 2019 Nov 7;20:822. doi: 10.1186/s12864-019-6225-9 (PMC6839148; doi:10.1186/s12864-019-6225-9)
Supplement: Supplementary file 1 — Additional file 1: Figure S1. Circle diagram of K. pneumoniae genome sequenced via Oxford Nanopore sequencing technology. Figure S2. Distribution of protein-coding genes predicted in 1567D strain. Figure S3. COG classification 1567D stain for the carbapenem-resistant K. pneumoniae. Figure S4. Distribution of K. pneumoniae genes annotated in GO term. Figure S5. Annotation of KEGG pathways in the carbapenem-resistant K. pneumoniae. Figure S6. Phylogenetic tree assessing the relatedness of the carbapenem-resistant K. pneumoniae strains in Fuzhou (purple) and in Hangzhou (green) to the reference genome database (blue). Table S1. Information of strains and patient diagnosis. Table S2. Phenotypes of 1566D, a.k.a., medical records of 1566P. Table S3. Phenotypes of 1567D, a.k.a., medical records of 1567P. Table S4. Phenotypes of 2035D, a.k.a., medical records of 2035P. Table S5. Phenotypes of 2036D, a.k.a., medical records of 2036P. Table S6. Phenotypes of 2037D, a.k.a., medical records of 2037P. Table S7. Phenotypes of 2038D, a.k.a., medical records of 2038P. Table S8. Phenotypes of 2039D, a.k.a., medical records of 2039P. Table S9. Illumina MiSeq sequencing yields. Table S10. Oxford Nanopore sequencing yields. Table S11. Detection and validation of SNPs in seven strains. Table S12. A total of 92 all-variation SNPs in seven strains. Red refers to 40 all-variation loci; Bold stands for 24 strain’s unique SNP loci. [file 12864_2019_6225_MOESM1_ESM.zip › Revision.SI-Supp Table 9-12_Supp Fig 1-6.docx]

**Additional file 1**

Xiaoling Yu, Wen Zhang, Zhiping Zhao, Chengsong Ye, Shuyan Zhou, Shaogui Wu, Lifen Han, Zhaofang Han^*^ and Hanhui Ye^*^

*** Correspondence to:**

Zhaofang Han, E-mail: zhaofang_han@foxmail.com.

Hanhui Ye, E-mail: 15960102808@163.com

This file includes:

**Figures S1 to S6**

**Tables S9 to S12** (**Tables S1-S8** are in separate Excel files)

**Supplementary Figures**

**Figure S1. Circle diagram of *K. pneumoniae* genome sequenced via Oxford Nanopore sequencing technology.**


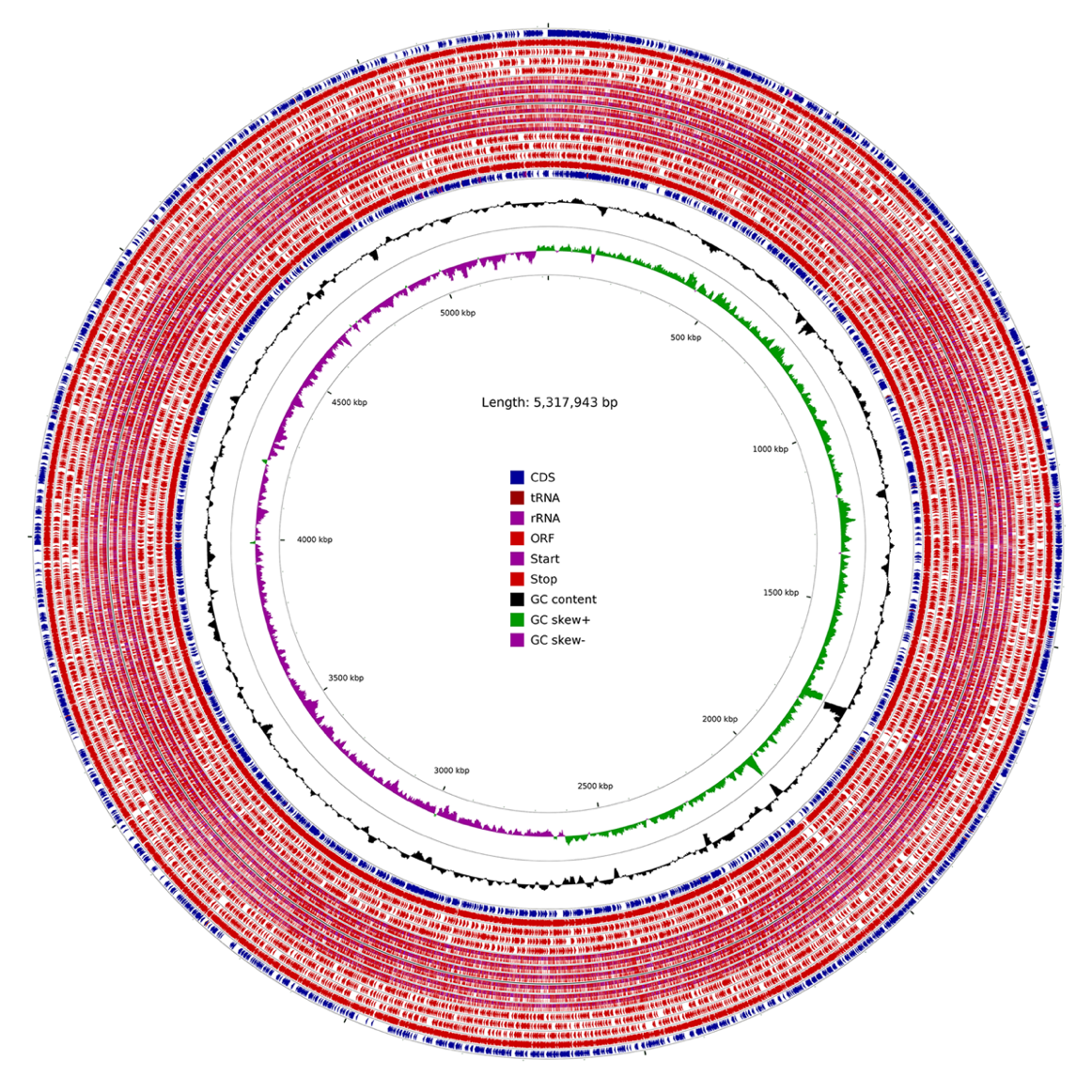


**Figure S2. Distribution of protein-coding genes predicted in 1567D strain.**


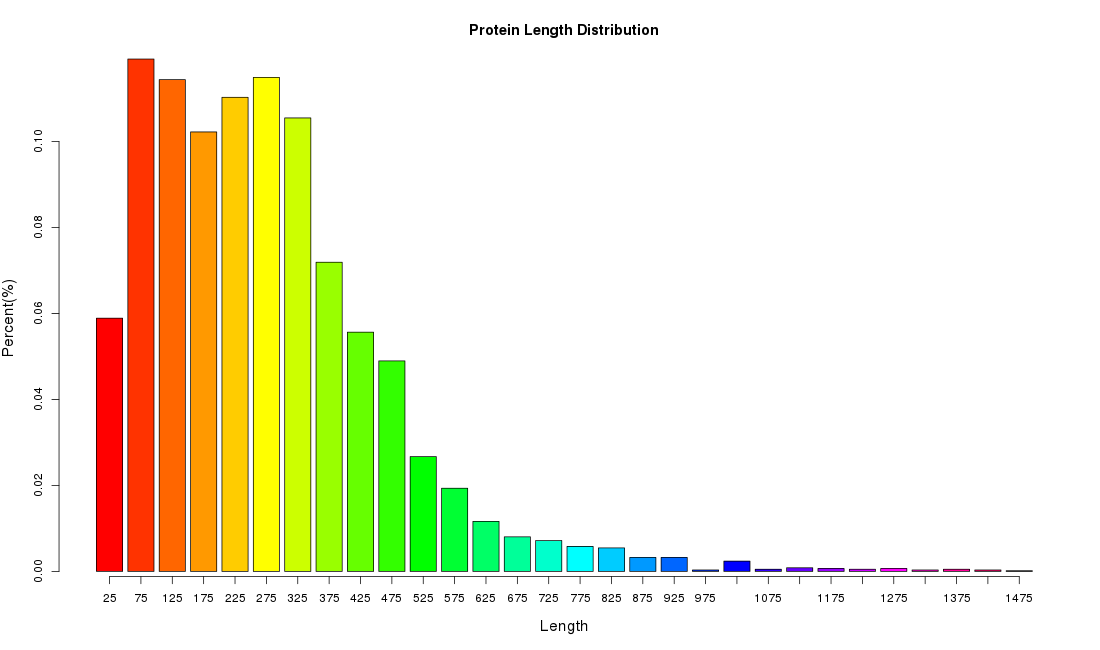


**Figure S3. COG classification 1567D stain for the carbapenem-resistant *K. pneumoniae.***


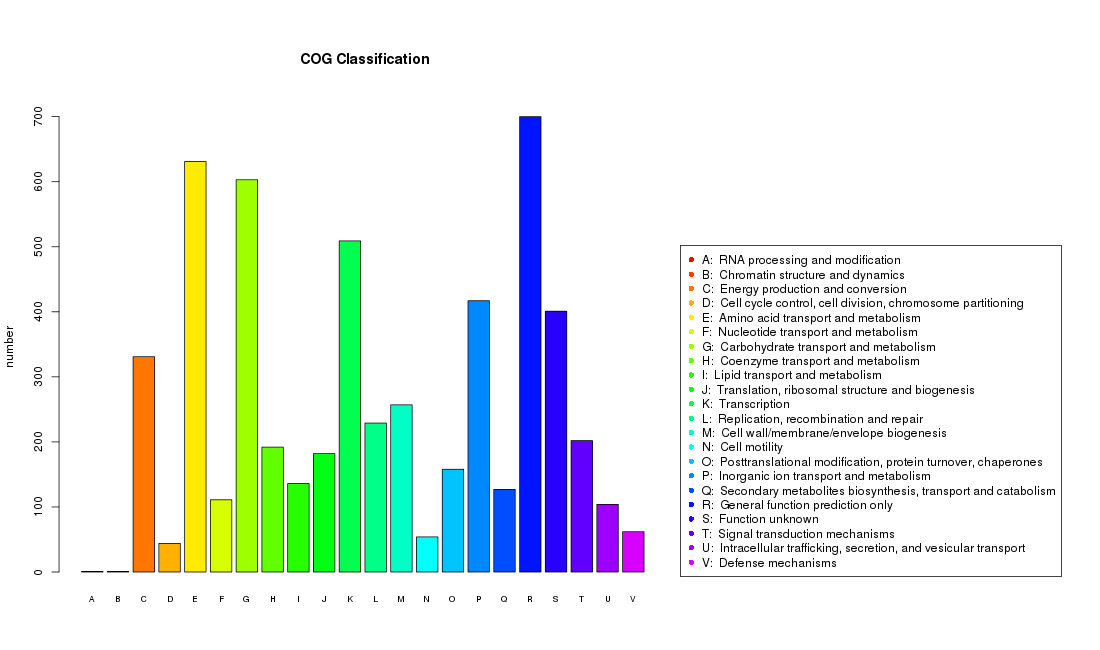


**Figure S4. Distribution of *K. pneumoniae* genes annotated in GO term.**


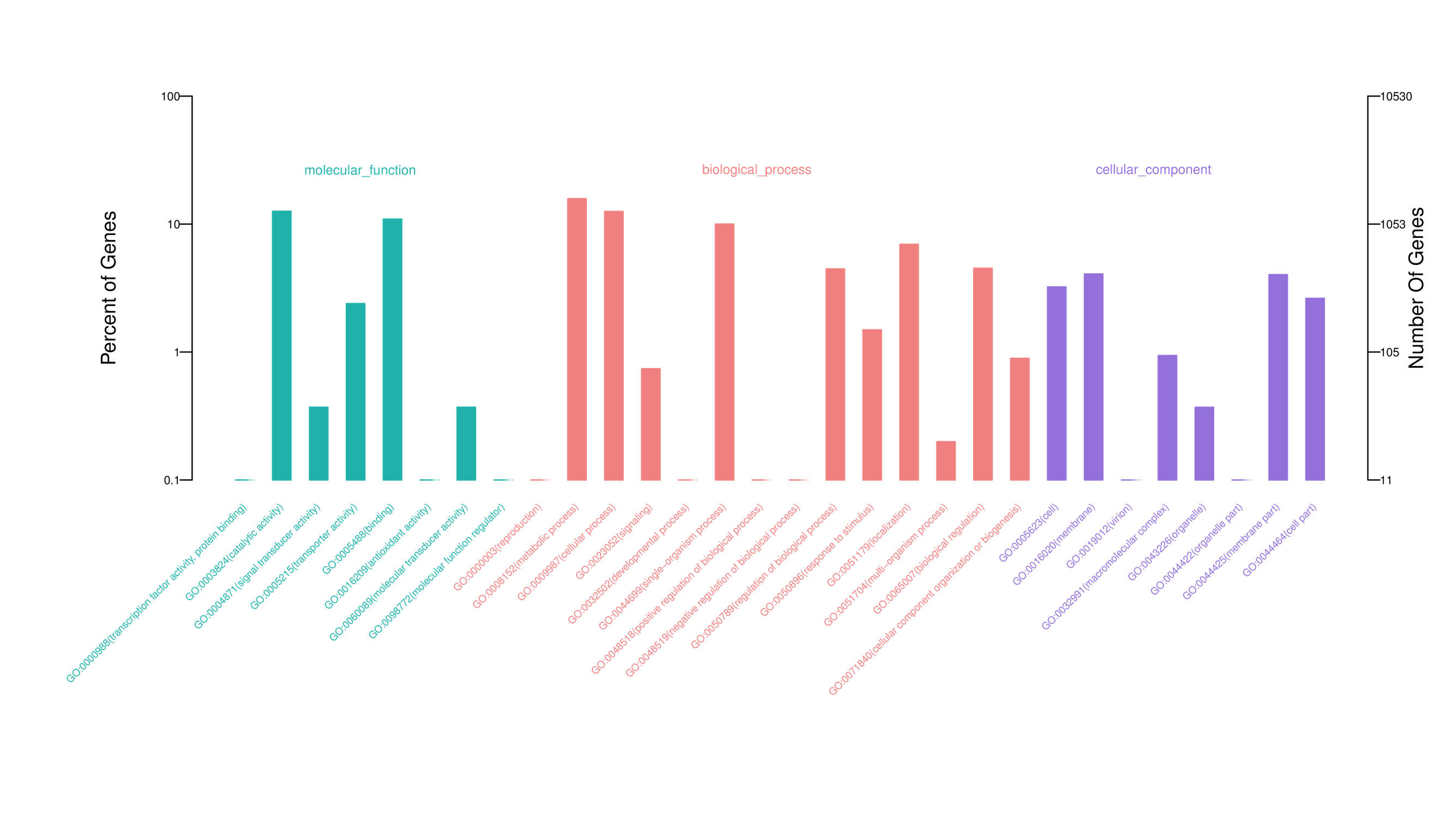


**Figure S5. Annotation of KEGG pathways in the carbapenem-resistant *K. pneumoniae*.**


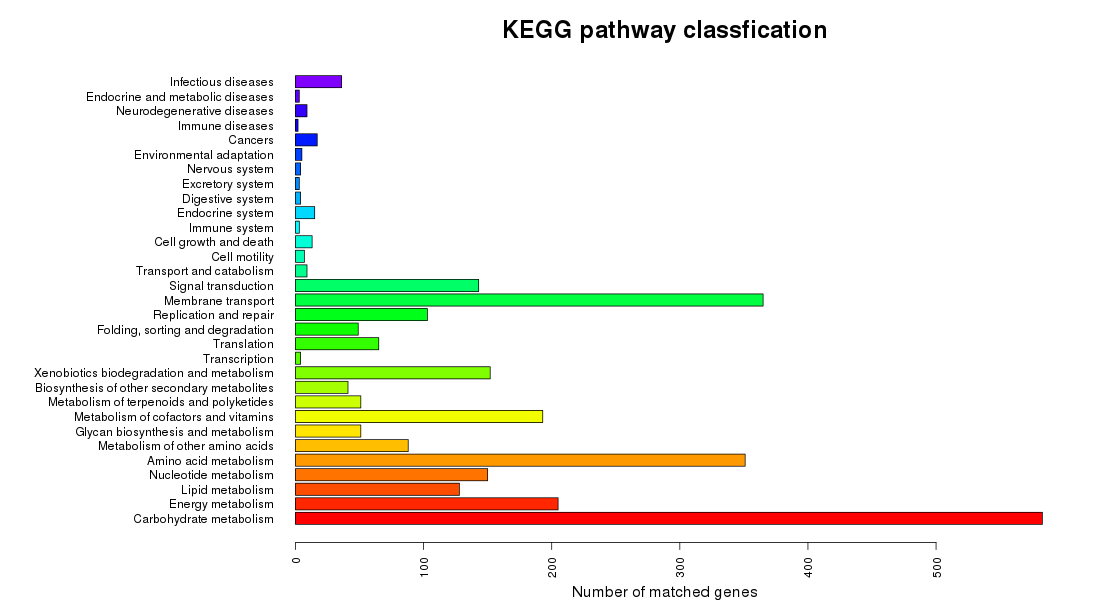


**Figure S6. Phylogenetic tree assessing the relatedness of the carbapenem-resistant *K. pneumoniae* strains in Fuzhou (purple) and in Hangzhou (green) to the reference genome database (blue).**

**Supplementary Tables**

All patients, except patient 1567P that was diagnosed as abdominal infection, were diagnosed as severe pneumonia or sufferred lung infections (**Table S1**). We give **Tables S2-S8** in detail to provide all patients’ treatment records as well as the phenotype measurement results and data. For instance, 2036P (**Table S5**) is an 83-year-old male patient. Previous to being admitted in our hospital, he was treated in another hospital. His medical record as of August 1^st^, 2017 showed that the urea nitrogen was 28.1 mmol/L, and creatinine was 429 μmol / L. His urinary red blood cell malformation rate was 56% high. Pulmonary CT indicate that there were nodules in his right upper lung, possibly being peripheral lung cancer. There were bilateral pleural effusion. He was hospitalized in our hospital from August 25^th^ to September 29^th^, 2017. For his and all other patients’ medical treatment records, please refer to **Tables S2-S8**.

All the eight strains (including the environmental isolate 2040D) were collected from the same floor (within the same ward) in our hospital (Table S1). Since the first CRKP (2035D) was reported on October 21, 2017, other CRKP’s were detected intermittently in October (2036D), November (2037D), and December (2038D and 2039D), 2017 in our hospital. It caused our attentions and we took interventions accordingly. After the above cases were reported, the microbiology room retained all the resistant strains since the hospital had a routine drug resistance monitoring. The CRKP’s that detected before October 21, 2017 were tracked and extracted from the strain bank, which were resuscitated. We did sequencing analysis for the additional two CRKP’s (1566D and 1567 D) together with the above CRKP’s. We analyzed them together in the article.

We collected the dining car that held lunches and dinners of the patients on that ward. We detected hands of the medical staff as well, and finally found the CRKP (2040D) on the dining car. No CRKP were detected elsewhere. With this source, the purpose of sequencing the bacteria car is to validate whether it has homology with other patients' CRKP. After taking the interventions, all the hospital beds and dining cars were pulled out to rinse with water, and the environment was disinfected.

**Tables S1-S8 are in separate Excel files and the table legends are as following:**

**Table S1. Information of strains and patient diagnosis.**

**Table S2. Phenotypes of 1566D, a.k.a., medical records of 1566P.**

**Table S3. Phenotypes of 1567D, a.k.a., medical records of 1567P.**

**Table S4. Phenotypes of 2035D, a.k.a., medical records of 2035P.**

**Table S5. Phenotypes of 2036D, a.k.a., medical records of 2036P.**

**Table S6. Phenotypes of 2037D, a.k.a., medical records of 2037P.**

**Table S7. Phenotypes of 2038D, a.k.a., medical records of 2038P.**

**Table S8. Phenotypes of 2039D, a.k.a., medical records of 2039P.**

**Tables S9-A12 are contained in the following of this document.**

**Table S9. Illumina MiSeq sequencing yields.**

| Sample | Total Reads | Raw Bases | Q20 Bases | Percentage (%) |
| --- | --- | --- | --- | --- |
| 1566D | 3,743,220 | 1,125,453,384 | 941,700,320 | 83.67 |
| 2035D | 4,782,134 | 1,320,197,359 | 1,163,174,482 | 88.11 |
| 2036D | 5,856,910 | 1,483,756,319 | 1,311,194,394 | 88.37 |
| 2037D | 6,624,804 | 1,657,988,991 | 1,460,537,145 | 88.09 |
| 2038D | 5,615,282 | 1,597,685,382 | 1,368,045,328 | 85.63 |
| 2039D | 5,641,658 | 1,439,029,823 | 1,263,496,926 | 87.80 |
| 2040D | 4,801,236 | 1,220,424,147 | 1,075,399,273 | 88.12 |

**Table S10. Oxford Nanopore sequencing yields.**

| Sample | Total reads | Total bases | Average length | Longest reads length | Reads N50 length |
| --- | --- | --- | --- | --- | --- |
| 1567D | 414,491 | 7,483,109,230 | 18,054 | 167,316 | 25,890 |

**Table S11. Detection and validation of SNPs in seven strains.**

| Isolates | SNP number | cSNP number | cSNP percent (%) | Validated SNPs | Validation ratio (%) |
| --- | --- | --- | --- | --- | --- |
| 1566D | 8,499 | 7,336 | 86.3 | 154 | 77.0 |
| 2035D | 7,506 | 6,424 | 85.6 | 147 | 73.5 |
| 2036D | 33,716 | 28,835 | 85.5 | 152 | 76.0 |
| 2037D | 7,488 | 6,399 | 85.5 | 154 | 77.0 |
| 2038D | 8,734 | 7,497 | 85.8 | 156 | 78.0 |
| 2039D | 8,624 | 7,424 | 86.1 | 155 | 77.5 |
| 2040D | 8,880 | 7,636 | 86.0 | 152 | 76.0 |

**Table S12. A total of 92 all-variation SNPs in seven strains.** Red refers to 40 all-variation loci; Bold stands for 24 strain’s unique SNP loci.

| Chromsome | Position | Ref | 1566D | 2035D | 2036D | 2037D | 2038D | 2039D | 2040D |
| --- | --- | --- | --- | --- | --- | --- | --- | --- | --- |
| NC_016845.1 | 1066275 | T | C/T | C/T | T | C/T | C/T | C/T | C/T |
| NC_016845.1 | 1066289 | T | G/T | T/G | G/T | G/T | G/T | G/T | G/T |
| NC_016845.1 | 1157834 | G | G/T | T/G | G | G | G | G | G |
| NC_016845.1 | 1309634 | T | G | G | G | G | G | G | G |
| NC_016845.1 | 1309841 | A | T | A | A/T | T | T | T | T |
| NC_016845.1 | 1309847 | G | A | A/G | A | A | A | A | A |
| NC_016845.1 | 1309865 | G | A | A | A | A | A | A | A |
| NC_016845.1 | 1309889 | C | T | T | T | T | T | T | T |
| NC_016845.1 | 1311041 | G | C | C | C | C | C | C | C |
| NC_016845.1 | 1311056 | C | A | A | **C** | A | A | A | A |
| NC_016845.1 | 1311080 | G | G | C/G | C | C | C | C | C |
| NC_016845.1 | 1311134 | G | A | A | A | A | A | A | A |
| NC_016845.1 | 1311558 | G | T | T | T | T | T | T | T |
| NC_016845.1 | 1322931 | G | T | T | **G** | T | T | T | T |
| NC_016845.1 | 1322970 | A | G | G | G | G | G | G | G |
| NC_016845.1 | 1323036 | C | T | T | **C** | T | T | T | T |
| NC_016845.1 | 1324201 | A | C | C | C | C | C | C | C |
| NC_016845.1 | 1324306 | C | T | T | T | T | T | T | T |
| NC_016845.1 | 1324519 | G | A | A | A | A | A | A | A |
| NC_016845.1 | 1325195 | A | G | G | G | G | G | G | G |
| NC_016845.1 | 1325197 | C | T | **C** | T | T | T | T | T |
| NC_016845.1 | 1325233 | A | G | **A** | G | G | G | G | G |
| NC_016845.1 | 1325260 | A | A | A | A | C | C | C/A | C |
| NC_016845.1 | 3049023 | T | C | C | C | C | C | C | C |
| NC_016845.1 | 3060510 | T | C | C | C | C | C | C | C |
| NC_016845.1 | 3060570 | C | T | T | T | T | T | T | T |
| NC_016845.1 | 3060768 | C | G | G | G | G | G | G | G |
| NC_016845.1 | 3061023 | C | T | T | T | T | T | T | T |
| NC_016845.1 | 3074462 | C | T | T | T | T | T | T | T |
| NC_016845.1 | 3090625 | T | C | C | C | C | C | C | C |
| NC_016845.1 | 3090874 | A | G | G | G | G | G | G | G |
| NC_016845.1 | 3091363 | A | G | G | G | G | G | G | G |
| NC_016845.1 | 3097079 | G | G/A | G/A | G/A | G/A | G/A | G/A | G/A |
| NC_016845.1 | 3103775 | A | A/G | A/G | G | A | A/G | A | A |
| NC_016845.1 | 3103779 | T | T | T | **G** | T | T | T | T |
| NC_016845.1 | 3121888 | T | T | T | **C** | T | T | T | T |
| NC_016845.1 | 3121980 | A | A | A | **G** | A | A | A | A |
| NC_016845.1 | 3122814 | C | C/T | T/C | C/T | C | C | C | C |
| NC_016845.1 | 3122948 | A | A | A | **G** | A | A | A | A |
| NC_016845.1 | 3122951 | T | T/C | T/C | **C** | C/T | C/T | T/C | T/C |
| NC_016845.1 | 3123167 | G | G | G | **A** | G | G | G | G |
| NC_016845.1 | 3123235 | G | G | G | **A** | G | G | G | G |
| NC_016845.1 | 3123236 | A | A/T | A/T | T | A/T | A | A | A |
| NC_016845.1 | 3123266 | G | G | G | **A** | G | G | G | G |
| NC_016840.1 | 2673 | A | G | G | G | G | G | G | G |
| NC_016840.1 | 2677 | C | T | T | **T/C** | T | T | T | T |
| NC_016840.1 | 2707 | C | T | **T/C** | T | T | T | T | T |
| NC_016840.1 | 2737 | T | G | G | **G/T** | G | G | G | G |
| NC_016840.1 | 2812 | T | A | A | A | A | A | A | A |
| NC_016840.1 | 2896 | C | T | T | T | T | T | T | T |
| NC_016840.1 | 2905 | C | T/C | T | T/C | C/T | C/T | T/C | T/C |
| NC_016840.1 | 2932 | C | G | G | G | G | G | G | G |
| NC_016840.1 | 3013 | C | A | A | A | A | A | A | A |
| NC_016840.1 | 3070 | C | A | A | A | A | A | A | A |
| NC_016840.1 | 3094 | A | T | T | T | T | T | T | T |
| NC_016840.1 | 3120 | A | C | C | C | C | C | C | C |
| NC_016840.1 | 3223 | G | A | A | A | A | A | A | A |
| NC_016840.1 | 3244 | G | C | C | C | C | C | C | C |
| NC_016840.1 | 3250 | C | T/C | T/C | C/T | C/T | T | T | T |
| NC_016840.1 | 3299 | G | C | C | C | C | C | C | C |
| NC_016845.1 | 3578729 | A | **A/G** | A | A | A | A | A | A |
| NC_016845.1 | 3578759 | T | **T/C** | T | T | T | T | T | T |
| NC_016845.1 | 3578876 | T | C/T | T/C | T/C | C/T | C/T | T/C | T/C |
| NC_016845.1 | 3578930 | T | C/T | T | T/C | C/T | C/T | T | T |
| NC_016845.1 | 3579029 | T | T | T/G | G/T | G/T | T | T | T |
| NC_016845.1 | 3579152 | G | A/G | A/G | **A** | G/A | G/A | G/A | G/A |
| NC_016845.1 | 3579305 | T | T | T | **C/T** | T | T | T | T |
| NC_016845.1 | 3579389 | T | T/G | T/G | G/T | G/T | G/T | G/T | G/T |
| NC_016845.1 | 3579394 | G | G | G | **A** | G | G | G | G |
| NC_016845.1 | 3579425 | G | G/A | G | G/A | G | G | G | G |
| NC_016845.1 | 3579517 | T | T/C | T/C | T/C | C/T | C/T | T/C | T/C |
| NC_016845.1 | 4873352 | G | G | G | **G/A** | G | G | G | G |
| NC_016845.1 | 5169646 | A | A/G | G | A/G | G | G | G/A | G |
| NC_016845.1 | 5169731 | G | T/G | G/T | G/T | G/T | G/T | G/T | G/T |
| NC_016845.1 | 5169737 | A | A/C | C | C | C | A/C | C/A | C/A |
| NC_016845.1 | 5169743 | A | T/A | A/T | **T** | A/T | A/T | T/A | T/A |
| NC_016845.1 | 5169844 | G | T/G | G | T | G | G | G/T | G/T |
| NC_016845.1 | 5169852 | T | C | C | C | C | C | C | C |
| NC_016845.1 | 5169857 | T | C/T | C/T | T/C | C/T | T | T | T |
| NC_016845.1 | 5169860 | G | G/C | G/C | C | C | C/G | G/C | G/C |
| NC_016845.1 | 5169865 | G | T/G | G | G | G/T | G | G/T | G/T |
| NC_016845.1 | 5169881 | G | T/G | T | T | T | G/T | G/T | G/T |
| NC_016845.1 | 5169890 | C | C/A | A | A/C | A | C/A | C/A | C/A |
| NC_016845.1 | 5169900 | C | T/C | C/T | C/T | C/T | C/T | C/T | C/T |
| NC_016845.1 | 5169938 | A | A/G | A/G | A/G | A/G | A/G | A/G | A/G |
| NC_016845.1 | 5169950 | A | C/A | C/A | A/C | A/C | A/C | C/A | C/A |
| NC_016845.1 | 5169980 | A | C | C | C | C | A/C | C | C/A |
| NC_016845.1 | 5169989 | A | A/G | G | A/G | G | A/G | A/G | A/G |
| NC_016845.1 | 5169992 | C | C/G | G | C/G | G | C/G | G/C | G/C |
| NC_016845.1 | 5170059 | C | C/T | T | T/C | T | C/T | T/C | T/C |
| NC_016845.1 | 5170096 | G | G | C | G | C | G | G | G |
| NC_016845.1 | 5170099 | A | G/A | G/A | A/G | **G** | A/G | G/A | G/A |
